# Metabolic fitness is decreased in monocytes of old individuals

**DOI:** 10.18632/aging.104025

**Published:** 2020-10-14

**Authors:** Kai Kisand, Pärt Peterson

**Affiliations:** 1 Molecular Pathology, Institute of Biomedicine and Translational Medicine, University of Tartu, Tartu 50411, Estonia

**Keywords:** monocytes, age, ribosomes, mitochondria, energy metabolism

Monocytes constitute the innate arm of immune defense, forming with macrophages and dendritic cells the first line of defense against invading pathogens [1]. With age, the host responses to infections and inflammatory stimuli decline, known as immunosenescence. This has been associated with the age-related chronic inflammatory processes, inflamm-aging, in which monocytes and monocyte-derived macrophages have an important role, as they are actively involved in inflammatory processes. Furthermore, their function strongly influences age-related diseases including atherosclerosis, sepsis, Alzheimer's disease, and has been more recently associated with severe COVID-19 patients.

The circulating pool of monocyte is divided into at least three subsets based on their expression of CD14 and CD16 surface markers. Classical monocytes, defined by CD14^+^CD16^−^, make up the majority (up to 95%) of the circulatory population, whereas the remaining subsets consist of non-classical CD14^lo^CD16^+^ and intermediate CD14^+^CD16^+^ monocytes [1]. Increasing age is associated with the overall shift of monocytes towards more inflammatory phenotype, and with an increased basal level of pro-inflammatory cytokines and their impaired *ex vivo* production in response to TLR ligands [2]. The monocyte population tends to expand with age, which is more evident in smaller subpopulations of non-classical and intermediate monocytes.

We recently studied CD14+ monocytes extracted from younger and older individuals to map their age-dependent changes by transcriptomic, epigenetic, and metabolomic profiling [3]. We found the monocytes from older persons to have a decline in gene expression of ribosomal proteins. The universal downregulation of the evolutionarily conserved machinery of protein synthesis on a level of ribosomal protein and rRNA has been noted in transcriptomic studies, albeit these changes are often difficult to monitor due to their constitutively high expression levels and rapid degradation [4]. The functional role of lowered protein synthesis remains enigmatic but has been associated with deregulated nutrient sensing and suggested to be a protective reaction to mitigate the age-linked changes as caloric restriction extends life-span and substantially decreases mRNA levels of ribosomal proteins through reduced mTOR activity [4].

The top downregulated genes also contained *PLA2G4B* and *ALOX15B* encoding enzymes in the arachidonic acid metabolism pathway, which we found to be in concordance with higher concentration of several phosphatidylcholines. This is likely related to monocyte activation, supporting the production of phospholipids and enhancing the secretion of proinflammatory cytokines [5]. However, the further analysis of specific phospholipid metabolites would be helpful to understand the exact impact of different subsets on blood plasma metabolite repertoire as age-dependent profiles have been reported.

In addition, we found the downregulation of the transcripts for mitochondrial proteins, which prompted us to study the mitochondrial changes in monocytes from old individuals. We found the age-related decrease in mitochondrial spare respiratory capacity, confirming the recent results by Pence and colleagues [6]. Furthermore, we noted an increase in molecular mass and lower mitochondrial membrane potential which per cell ratio was significantly lower in both classical and nonclassical monocytes. The link between the mitochondrial changes and proinflammatory phenotype in aged monocytes remains an intriguing idea as mitochondrial dysfunction and oxidative stress can activate inflammasomes via NLRP3 with potential impact on age-related diseases such as atherosclerosis and Alzheimer’s disease [7].

In our transcriptome screen, we observed the upregulation of the PDK4 gene, which has a key role in maintaining normal glucose levels and which inhibits the formation of acetyl-coenzyme A from pyruvate and down-regulates aerobic respiration. This suggested that in response to age-related inflammatory signals the monocytes from old individuals may shift glucose metabolism from oxidative phosphorylation toward glycolytic ATP production seen in cellular activation [5]. This was also in agreement with higher uptake of the glucose analog 2‐NBDG in aged individuals.

**Figure 1:**
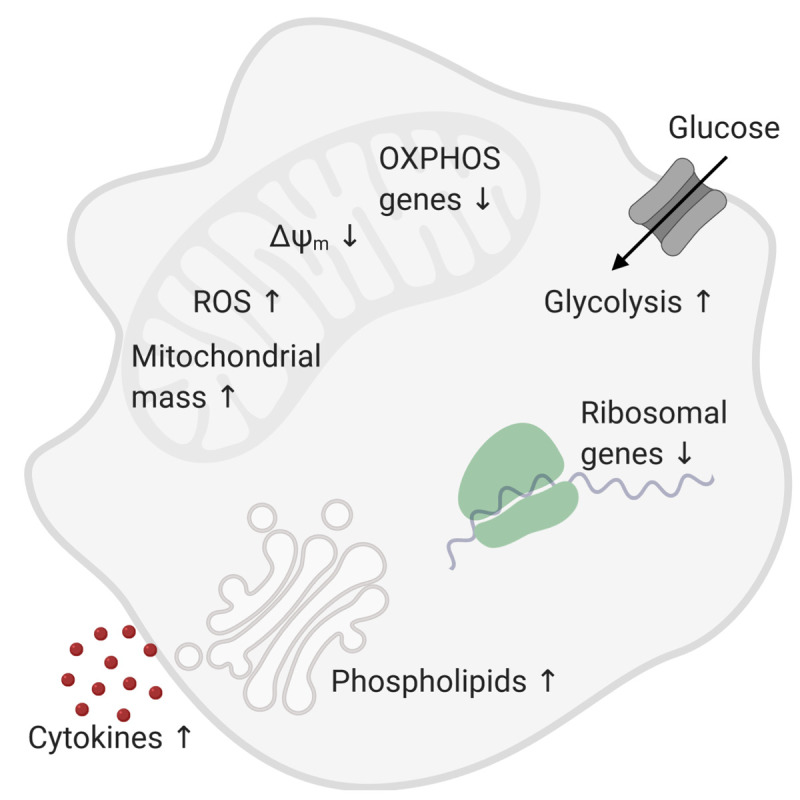
Metabolic changes in monocytes of old individuals.

Together with the decreased expression of genes related to oxidative phosphorylation, decreased mitochondrial potential, and reduced spare respiratory capacity, our findings suggested that the balance of energy metabolism in monocytes has tipped with age from oxidative phosphorylation toward aerobic glycolysis. Thus, the metabolic fitness of monocytes from aged persons seems to be impaired with decreased mitochondrial respiratory reserve and restricted capacity to utilize additional glucose (Figure 1).

The CD14+, CD16+ monocytes producing high levels of pro-inflammatory IL-6 and TNFa are increased in severe COVID-19 patients admitted to intensive care units (reviewed in [8]). The mechanisms activating monocytes and/or macrophages in COVID-19 and their contribution to COVID-19 pathophysiology remain to be clarified but they could be enhanced by age-related changes contributing to mitochondrial dysfunction, oxidative stress or inflammasome activation. The clarification of extent and contribution of age-related changes in monocytes and monocyte-derived macrophages to COVID-19 disease severity would be critical for the development of immunomodulatory strategies to treat COVID-19 patients.
